# P-514. Enhancing the Pediatric Primary Care Provider’s Role in Adolescent HIV Prevention

**DOI:** 10.1093/ofid/ofae631.713

**Published:** 2025-01-29

**Authors:** Claudia P Vicetti Miguel, Margaret Thew, Wendi Ehrman, Lia Mojica

**Affiliations:** Medical College of Wisconsin, Wauwatosa, Wisconsin; MCW, Milwaukee, Wisconsin; Medical College of Wisconsin 8701 W. Watertown Plank Rd, Milwaukee, WI 53226, Milwaukee, Wisconsin; Medical College of Wisconsin, Wauwatosa, Wisconsin

## Abstract

**Background:**

Youth with a history of bacterial sexually transmitted infections (STIs) are at increased risk of HIV acquisition and should be counseled on HIV Pre-Exposure Prophylaxis (PrEP). PrEP counseling has been associated with high acceptability rates in primary care, but most pediatric providers do not discuss PrEP during clinic visits. We conducted a quality improvement project at primary care clinics affiliated with a tertiary not-for-profit children’s hospital serving the city of Milwaukee. The objective of this project is to increase rates of counseling on HIV testing and PrEP during primary care visits around an STI diagnosis, as part of a larger institutional initiative to increase PrEP utilization in at-risk youth.

**Figure:**
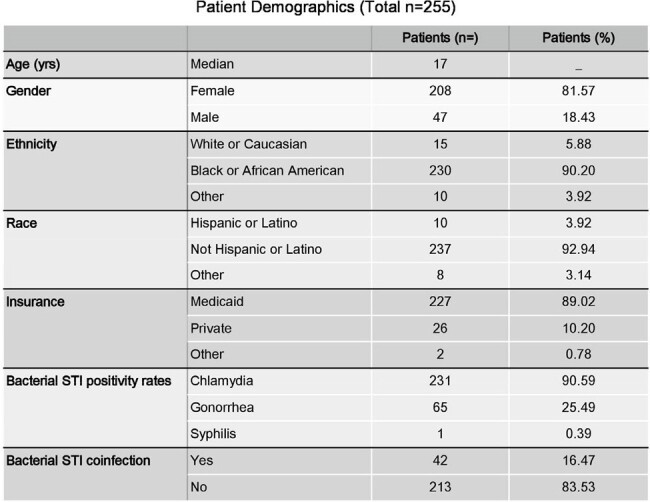

**Methods:**

Rates of HIV testing and PrEP counseling were monitored monthly during baseline (July 2022-June 2023) and post-intervention periods (July 2023-June 2024) using Plan-Do-Study-Act (PDSA) cycles tracked via a statistical process control chart.

We included well child and sexual health-related office visits in youth aged 12 and older that occurred within 3 months of a bacterial STI diagnosis (gonorrhea, chlamydia, or syphilis). Interventions consisted of a clinician educational session, a prompt in the electronic health record (EHR) for missing HIV tests and a communication to the primary care provider containing internal PrEP resources following a patient’s STI diagnosis.

**Figure:**
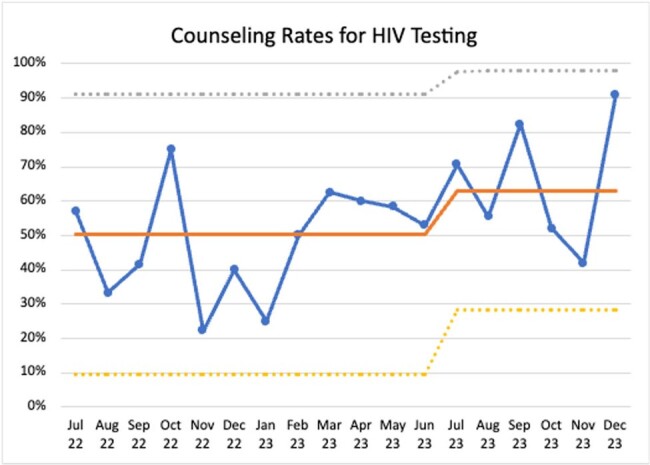

**Results:**

From July 2022 to December 2023, we identified 255 primary care visits around an STI diagnosis (90.59% chlamydia, 25.49% gonorrhea, 0.39% syphilis and 16.47% co-infections). Patient characteristics are summarized in Table 1. Interim analysis showed counseling rates on HIV testing increased from 50% to 63% (Figure 1) and PrEP counseling from 5% to 15% (Figure 2) through December 2023. Data will continue to be measured through June 2024.

**Figure:**
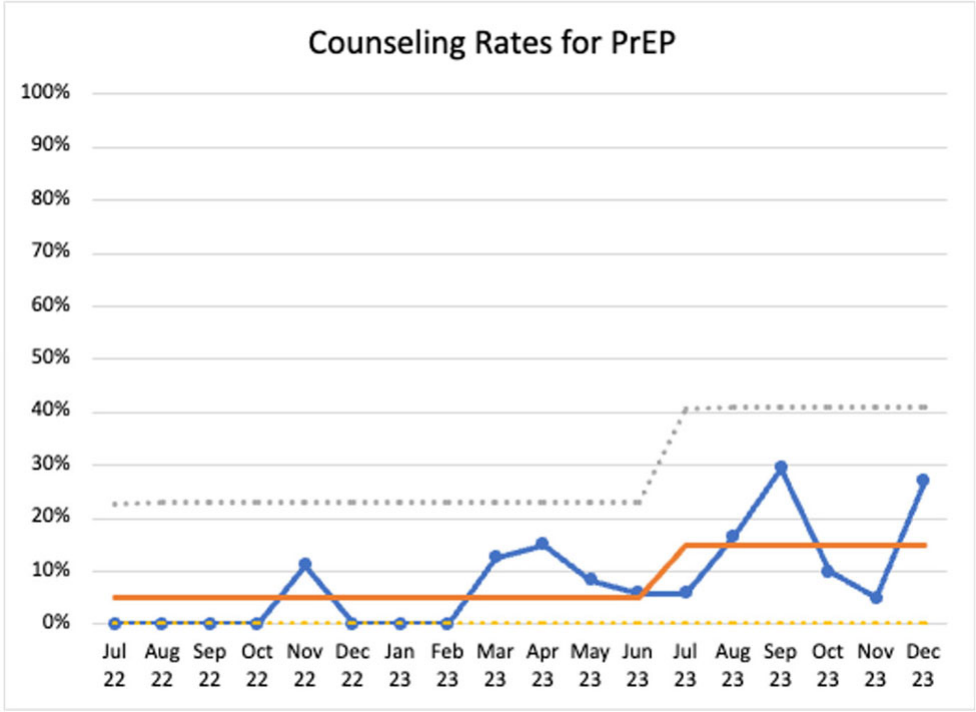

**Conclusion:**

For the first 6 months of our project, clinician education combined with an EHR prompt and provider communications containing PrEP resources have resulted in a modest increase in counseling rates on HIV testing and PrEP for at-risk youth during primary care visits. These initial interventions demonstrate a promising start towards enhancing HIV prevention discussions in pediatric primary care.

**Disclosures:**

**All Authors**: No reported disclosures

